# Supplementary material for: Genome-scale detection of positive selection in nine primates predicts human-virus evolutionary conflicts
Source: Nucleic Acids Res. 2017 Aug 11;45(18):10634–48. doi: 10.1093/nar/gkx704 (PMC5737536; doi:10.1093/nar/gkx704)
Supplement: Supplementary Data [file gkx704_supp.zip › nar-01319-n-2017-File007.pdf]

SUPPLEMENTARY INFORMATION

for

Genome-scale detection of positive selection in 9 primates predicts human-virus evolutionary conflicts

Robin van der Lee[1,*,^], Laurens Wiel[1,2], Teunis J.P. van Dam[1,#], Martijn A. Huynen[1]

[1]Centre for Molecular and Biomolecular Informatics, [2]Department of Human Genetics, Radboud Institute for Molecular Life Sciences, Radboud university medical center, Nijmegen, The Netherlands

[*]To whom correspondence should be addressed.
Tel: +1 604 875 2345 x5273
Email: robinvanderlee@gmail.com

[^]Present address: Centre for Molecular Medicine and Therapeutics, Department of Medical Genetics, BC Children's Hospital Research Institute, University of British Columbia, Vancouver, BC, Canada
[#]Present address: Theoretical Biology and Bioinformatics, Department of Biology, Faculty of Science, Utrecht University, The Netherlands

Supplementary Figures

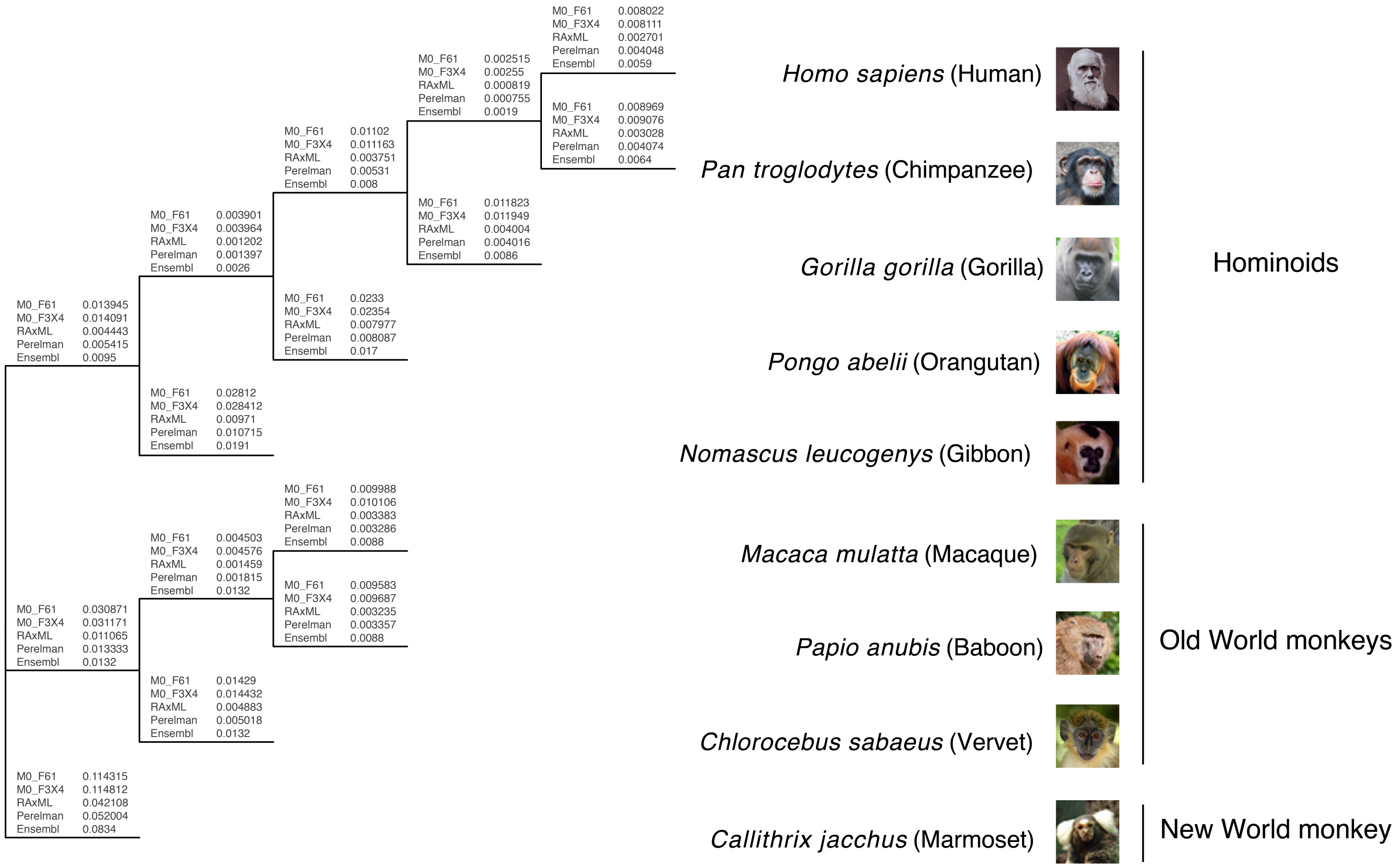

Figure S1. Phylogenetic trees of the nine simian primates selected for the analyses. Plotted on top of the well-supported primate topology are branch lengths of five different phylogenetic trees. (**M0_F61**, **M0_F3X4**) Protein coding-based reference phylogenetic trees used in all ML analyses. These trees were calculated using the codeml M0 evolutionary model under the F61 (M0_F61, same tree as in Figure 2) or F3X4 (M0_F3X4) codon frequency parameters on a concatenated alignment of 11,096 protein-coding, one-to-one orthologous genes of the nine primates studied. Other statistics: [M0_F61] kappa (ts/tv) = 3.91981, dN/dS =0.21341, dN=0.0477, dS = 0.2235; [M0_F3X4] kappa (ts/tv) = 4.15152, dN/dS =0. 21682, dN=0.0484, dS = 0.2231. (**RAxML**) Maximum likelihood phylogenetic tree of the same concatenated alignment, inferred using nucleotide rather than codon evolutionary models. (**Perelman**) Nine primates extracted from a 186-primate phylogeny based on genomic regions of 54 primate genes (consisting half of noncoding parts) from Perelman et al. (1). (**Ensembl**) Adapted from the full species tree of Ensembl release 78 (December 2014), which is based on the mammals EPO whole-genome multiple alignment pipeline (2). Branch lengths are in nucleotide substitutions per site, with 'sites' being codons in (M0_F61, M0_F3X4) and nucleotides in (RAxML, Perelman, Ensembl). Species pictures were taken from Ensembl and **Table S1**.

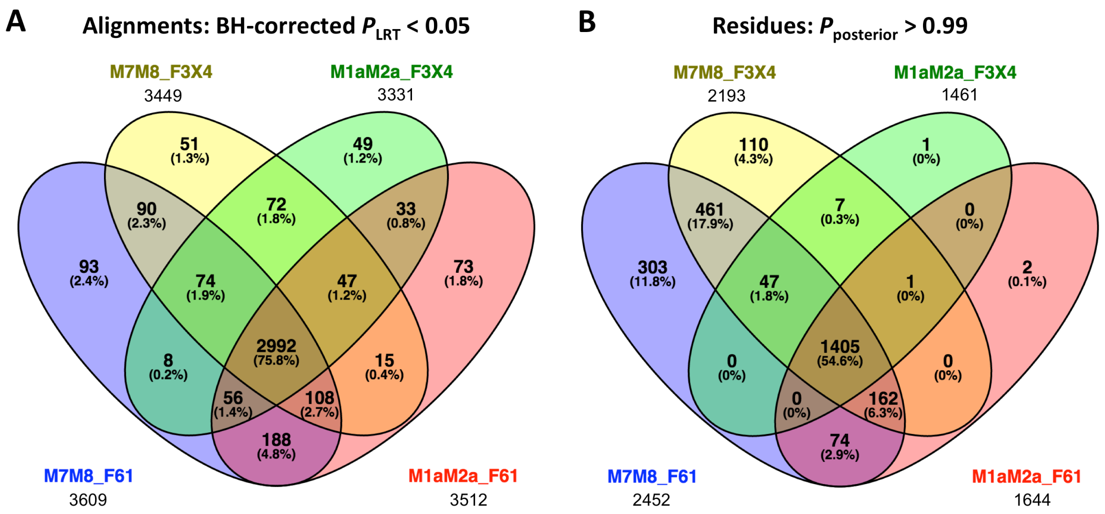

Figure S2. Overlaps between positive selection predictions from four evolutionary model parameters combinations. Apparent Positively Selected Genes (aPSG, **A**) and Residues (aPSR, **B**). Only for significant aPSG did we collect aPSR from the site-specific codeml predictions. See **Methods**. Venn diagrams created using Venny (3).

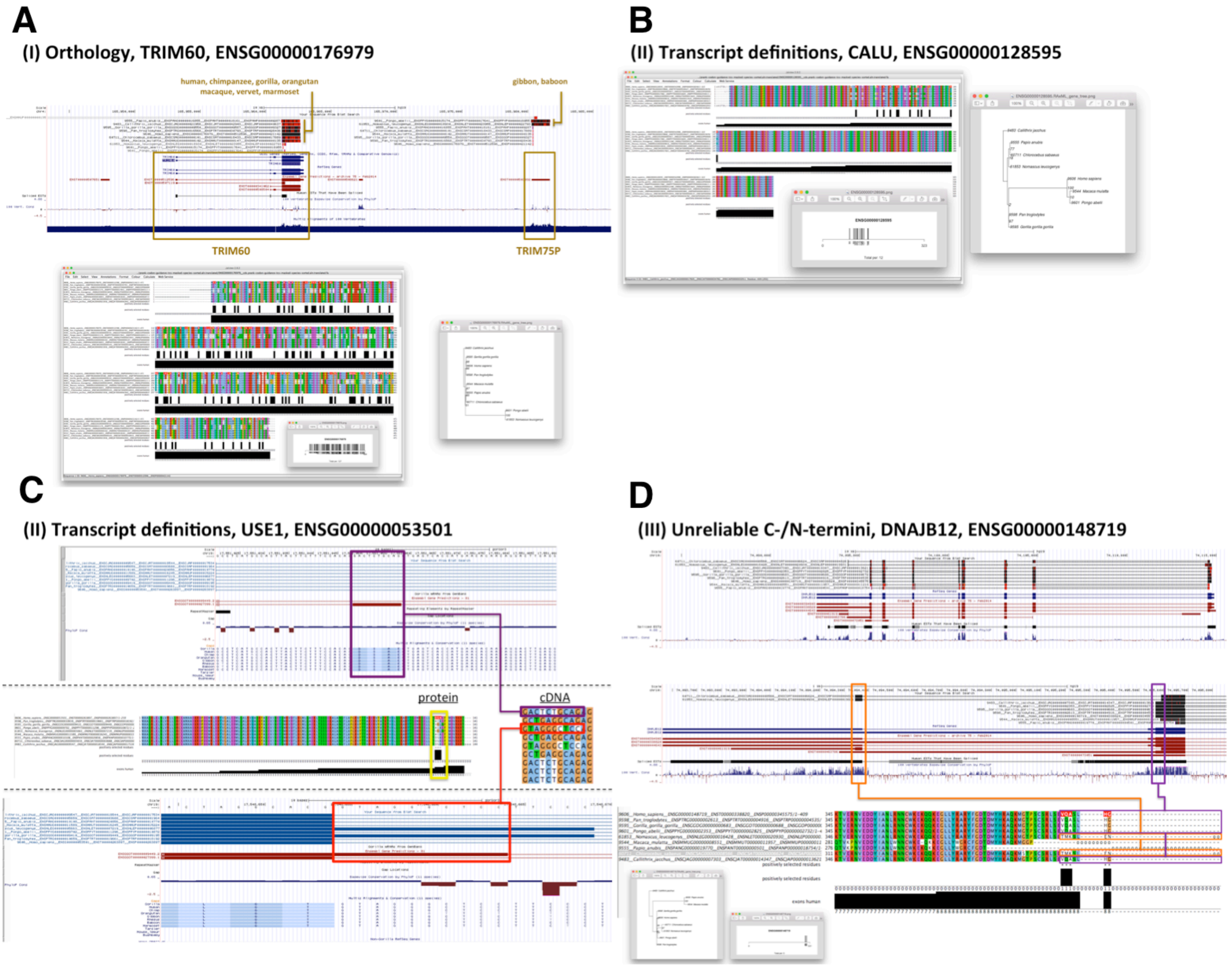

Figure S3. Examples of positive selection artefacts. (**A**) A type-I problem (orthology) involving the clustering of outparalogs *TRIM60* and *TRIM75*. The distinct sets of sequences differ across their whole length, leading to artificially high substitution rates across the whole alignment. See also **Figure 3B**. (**B**) A type-II problem (transcript definitions) involving mutually exclusive, tandem duplicated exons in the *CALU* gene. All aPSR locate to a single mapped exon. See also **Figure 3C**. (**C**) A type-II problem (transcript definitions) involving three distinct sets of sequences for the *USE1* gene across the primate species. These sequence sets originate from different gene models in the different species, some of which include a small exon (top genome browser screenshot), while others have an extended 3' exon boundary (bottom). All aPSR locate to the same small region in the protein. (**D**) A type-III problem (unreliable C-terminus) in the *DNAJB12* gene. In some species the gene model includes a slightly longer last coding exon, while in others it features a small coding region within the region that is part of the UTR in others. All aPSR locate to the small region at the alignment C-terminus. Panels further show alignments that are codon-based, masked and translated, as well as their gene trees. The lower black blocks under the alignments indicate exon coordinates mapped to the protein alignments, while the black bars above the exon blocks indicate the predicted aPSR. Barcodes indicate the distribution of aPSR across the sequences. UCSC Genome Browser (4) screenshots show BLAT alignments of cDNA sequences of the nine primates (black tracks).

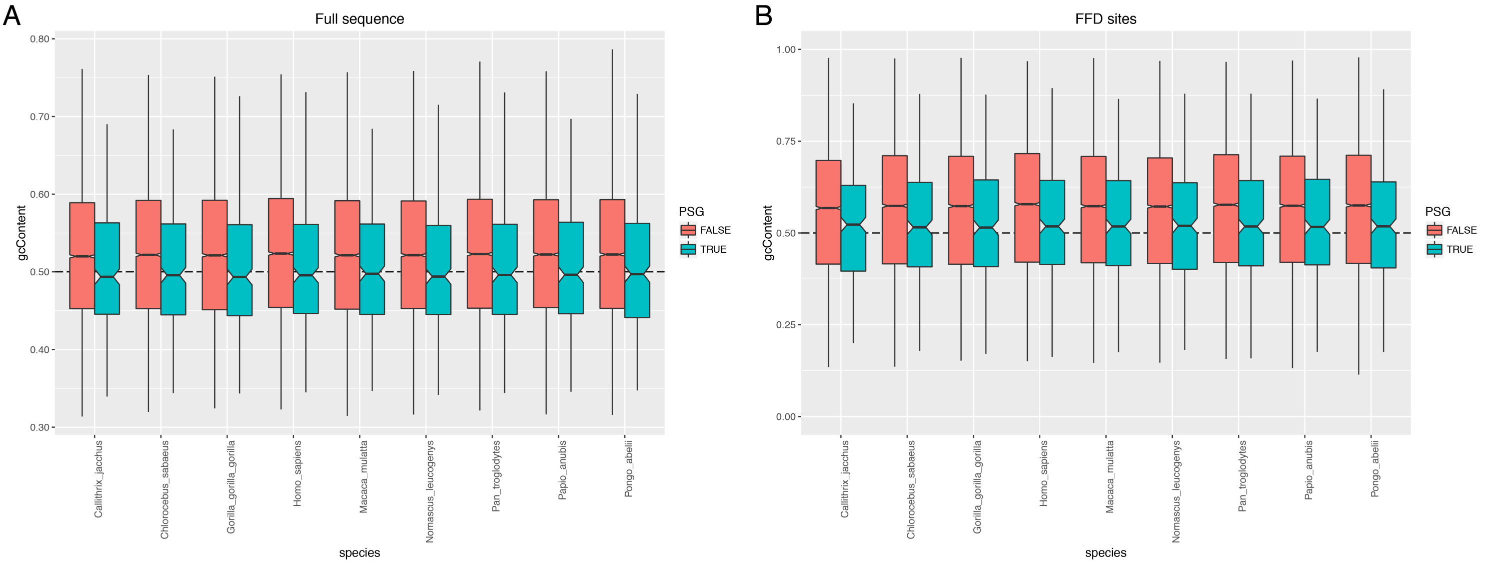

Figure S4. Comparison of the GC contents of PSG (N=331) and non-PSG (N=10,839) across the nine primates studied. GC content was calculated as the fraction of nucleotides that are G or C across the full coding sequences (**A**) or across all fourfold degenerate (FFD) sites (ACN CCN CGN CTN GCN GGN GTN TCN; **B**). Note that Y-axes have different limits in (A) and (B). Boxplots in both (A) and (B) include all data points, i.e. there are no 'outliers'. FFD sites tend to have slightly higher GC content than the full coding sequences, but PSG have lower GC content than non-PSG. The general lack of housekeeping functions in our PSG may be the cause of the lower GC content of our PSG compared to other genes (i.e. housekeeping-like genes tend to have high GC content).

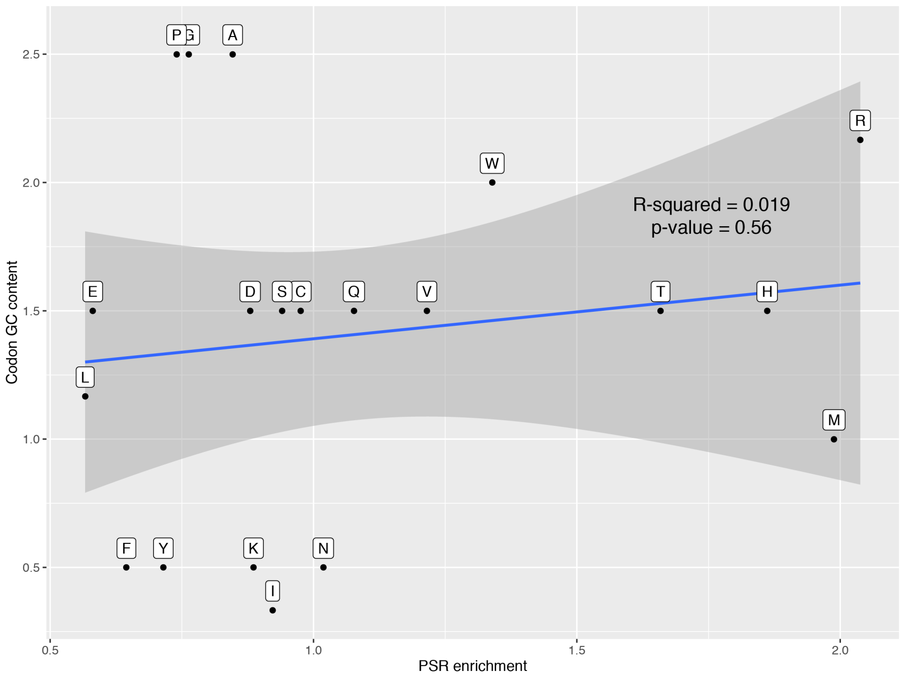

Figure S5. Correlation between PSR amino acid enrichment scores and codon GC content. Codon GC content was calculated as the average GC content of all codons for a particular amino acid. Plot shows the linear regression line with 95% CI. See **Figure S7** for the PSR enrichment scores.

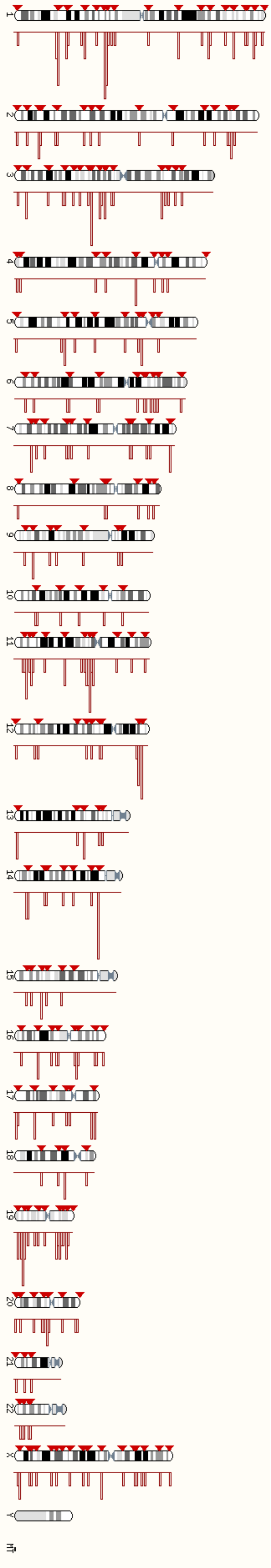

Figure S6. Distribution of PSG across the human genome and chromosomes. Individual PSG are marked by the red triangles to the left of the chromosomes; bars to the right indicate total numbers of PSG across the chromosomes. The numbers of PSG per chromosome are not significantly different from the expected numbers based on the total number of protein-coding genes per chromosome (*P* ≈ 0.1, chi-squared test). Not in the figure: the PSG are distributed roughly equally across the different DNA strands (169/331[51%] are on the minus strand, 162/331 [49%] are on the plus).

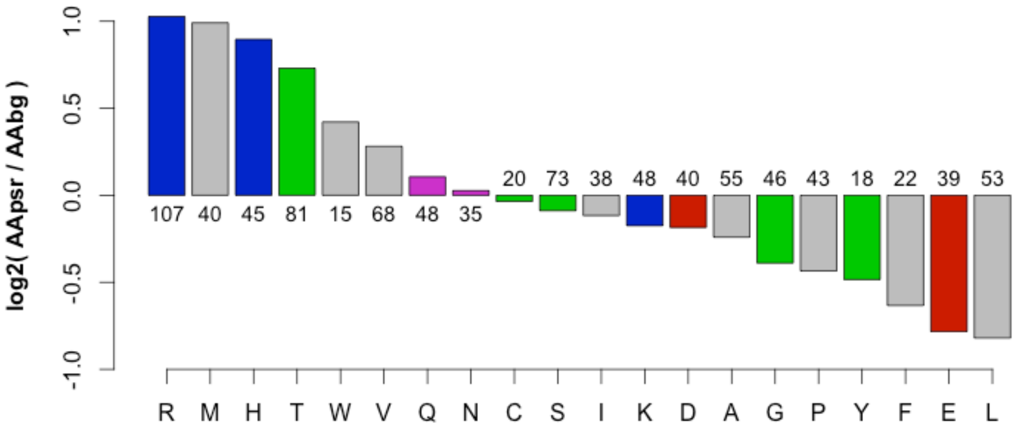

Figure S7. Occurrences of amino acids in the human sequence at the position of a PSR (positively selected site in the alignment). The y-axis represents enrichment scores comparing the PSR amino acid distribution (absolute numbers indicated) to a background distribution of amino acid occurring in all human sequences (11,096) used for our evolutionary analyses: $ES_{AA} = {}^{2}\log(\text{ fraction}(AA_{PSR}) / \text{fraction}(AA_{background}))$. Amino acid colors: blue = basic; red = acidic; green = polar; purple = neutral; gray = hydrophobic.

Supplementary Tables

Table S1. Overview of genomes used in the analysis, taken from Ensembl release 78 (December 2014)(2).

**Common name**	**Scientific name**	**NCBI Taxonomy ID**	**First genome (publication) available**	**Picture[#]**
Human	*Homo sapiens*	9606	2001 (5, 6)	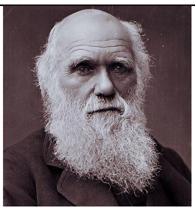
Chimpanzee	*Pan troglodytes verus*	9598	2005 (7)	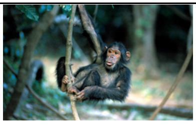
Gorilla	*Gorilla gorilla gorilla*	9595	2012 (8)	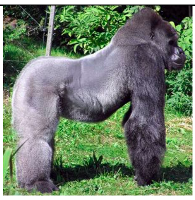
Orangutan	*Pongo abelii*	9601	2011 (9)	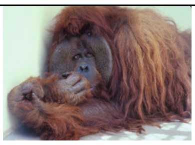
Gibbon	*Nomascus leucogenys*	61853	2014 (10)	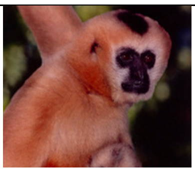
Macaque	*Macaca mulatta*	9544	2007 (11)	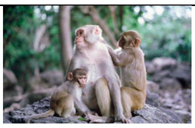
Baboon	*Papio anubis*	9555	2012 (https://www.hgsc.bcm.edu/non-human-primates/baboon-genome-project)	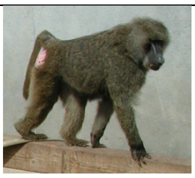
Vervet (African Green Monkey)	*Chlorocebus sabaeus*	60711	2014 (http://www.genomequebec.mcgill.ca/compgen/vervet_research/genomics_genetics/)	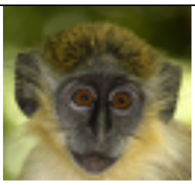
Marmoset	*Callithrix jacchus*	9483	2014 (12)	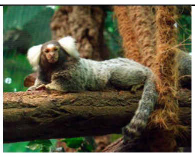

[#]Species pictures were taken from the UCSC Genome Browser (4), Ensembl (2) and https://commons.wikimedia.org/wiki/File:Charles_Darwin_photograph_by_Herbert_Rose_Barraud,_1881.jpg

Table S10. Analysis of the 19 PSG from our evolutionary analyses that encode proteins localized to the mitochondrion (13).

**Positively selected gene**	**Protein interacts with mitochondrial-encoded molecule**	**Positively selected residue interacts with mitochondrial-encoded molecule**
TRIT1	Yes, tRNA	No
PTCD2	Yes, mRNA, possibly cytochrome b mRNA (14)	No structure data
MAVS	No, but MAVS is not in the mitochondrial matrix	-
COA1	Yes, COX1 (15)	No structure data
FOXRED1	Yes, ND4 (16)	No structure data
MRPS30	Yes, ribosomal RNA	Yes, in PDB 3J7Y
FAM162A	Not known	No structure data
MTIF3	No direct evidence; does interact with the mitochondrial ribosome	No structure data
NDUFA10	Yes, ND2 (17)	No
LRPPRC	Yes, mRNAs (18)	Yes, positively selected residue Arg1139 in *LRPPRC* aligns with position 35 of the PPR repeat as predicted with TPRpred (19). Position 35 of the repeat is known to interact with RNA, e.g. in PPR10 (PDB 4M59).
FARS2	Yes, tRNA	No
PDSS1	Likely not, Ubiquinone synthesis	No interaction with a mitochondrial-encoded molecule
GUF1	Yes (20)	No structure data
TFB2M	Yes (21)	No structure data
TMEM126A	Yes, ND2, ND3, ND4L, ND6 (16)	No structure data
TMEM126B	Not known	No structure data
TRMT10C	Yes, tRNA	No structure data
TMEM186	Not known	No structure data
OXLD1	Not known	No structure data

Table S2. 11,170 clusters of one-to-one orthologous genes in the nine primates.
(text file)

Table S3. 416 apparent Positive Selected Genes (aPSG) systematically inspected for artefacts.
(text file)

NB: Each aPSG may be affected by more than one type of problem. Artefacts were classified into five classes (see **Figure 3** for an overview):

1. Alignments of sets of sequences that differ across their whole length. Probably a gene clustering/orthology inference problem, often seems to result in an alignment of paralogs (since the sequences are similar enough to be aligned, but are divergent enough to be clearly marked as not orthologous). Often identified because: 1) very many PSR are detected across the whole length of the alignment, 2) the distinct sets of gene sequences are usually not distributed according to the species phylogeny, 3) the gene tree has one (in the case of a single sequence being very different from the rest) or a group of unusually long branches.
   1a. Each sequence set is represented by multiple sequences
   1b. A single sequence is very different from all the others

2. Alignment of sets of sequences that differ in one or more (distinct though often similar) exons. Probably a gene model/alternative transcript problem, often seems to be the result of exon duplication and divergence (since the exons are similar enough to be aligned, but are divergent enough to be clearly identified as not orthologous). Occasionally can also be the result of different exon/intron boundaries. Often identified because: 1) all PSR locate to a single exon, 2) the distinct sets exon sequences are usually not distributed according to the species phylogeny, 3) the gene tree has a group of unusually long branches (although this is only clearly visible if the exon is long enough).

3. Terminal sequence region(s) are unreliably aligned, often concurrent with different terminal exon sequence sets. Not always sure how this is caused; sometimes it seems a gene model problem where - in the case of a C-terminus problem - for some sequences instead of adding the 'real' last exon, the second-to-last exon is extended (most likely until the first stop codon?); similarly – in case of a N-terminus problem – for some sequences instead of including the 'real' first exon, the second exons is longer at the N-terminus / 5'-end (most likely until the first start codon?). Often identified because: 1) the terminal regions contain very many, and often all, PSR detected for the alignment, 2) the sequence divergence in these regions is unrealistically high.
   3a. N-terminus
   3b. C-terminus

4. Unreliable alignment surrounding the PSR. Often seems to be caused because of small inconsistent gene models / alternative exons / exon boundaries across the different sequences that cause parts of the sequences and alignment to be masked and generally remain hard to align; regions are often very small which seems to cause the aligner to try to fit them somewhere rather than making clean inserts. Often identified because: 1) gaps or 2) masked residues surround the relevant region in the alignment.

5. Other. See individual cases.

Table S4. 331 PSG: human genes with strong statistical evidence for positive selection across nine primates.
(text file)

Table also shows statistics for all four tested combinations of ML evolutionary model parameters, including LRT tests and (corrected) *P* values (see **Methods**).

Table S5. 934 PSR: positively selected residues (codons) detected in the 331 PSG.
(text file)

Table also shows statistics for all four tested combinations of ML evolutionary model parameters, including Bayesian posterior probabilities and estimated omegas (see **Methods**).

Table S6. Function enrichment analysis of PSG using Babelomics (22).
(excel file)

Table S7. Function enrichment analysis of PSG using DAVID (23).
(excel file)

Table S8. Overlaps of the PSG with various virus-host interaction, immunity, and other datasets.
(text file)

Table S9. Known (antiviral) immunity genes detected to be under positive selection in small-scale evolutionary studies.
(excel file)
Some studies provided experimental support for the importance of the detected positively selected codons. Tab1: overview of genes. Tabs 2-5: detailed analyses of *TRIM5*, *MAVS*, *BST2* [tetherin], *SAMHD1*).

Table S11. ExAC human variation statistics for primate PSG and non-PSG.
(text file)

Supplementary Files

Available at
https://github.com/robinvanderlee/positive-selection/tree/master/Supplementary_data_and_material

Original coding and proteins sequences used in our analyses

- Protein-coding DNA sequences
  `cds_sequences_11170_genes.tar.gz`
  (11,170 files, 9 sequences per file, 1 per primate)
- Protein sequences
  `protein_sequences_11170_genes.tar.gz`
  (11,170 files, 9 sequences per file, 1 per primate)

PRANK alignments, filtered and masked using GUIDANCE and TCS

- Protein-coding DNA sequence alignments
  `prank-codon-guidance-tcs-masked-species-sorted.aln__11096.tar.gz`
  `N`: undetermined nucleotides
  `n`: masked nucleotides
- Translated alignments
  `prank-codon-guidance-tcs-masked-species-sorted.aln.translated__11096.tar.gz`
  `X`: undetermined amino acids
  `o`: masked amino acids
- Mapping tables of the alignment column to the position in the human sequence
  `prank-codon-guidance-tcs-masked-species-sorted.aln.translated.mapping-table__11096.tar.gz`
  Column 1: human amino acid
  Column 2: columns number of the alignment
  Column 3: human sequence position
- Concatenation of the 11,096 protein-coding DNA sequence alignments
  `concatenated_alignment__11096_genes__9primates__cds.prank-codon-guidance-tcs-masked-species-sorted.aln.fa.gz`
  `concatenated_alignment__11096_genes__9primates__cds.prank-codon-guidance-tcs-masked-species-sorted.aln.phy.gz`

Visualization and quality control of alignments and positive selection profiles

- Barcode plots of positive selection profiles
  - Just the curated set of 331 positively selected genes (PSG): `barcode_plots__331_PSG.tar.gz`
  - All 416 original, apparent positive selected genes (aPSG): `barcode_plots__416_aPSG.tar.gz`
- Jalview alignment annotations
  `jalview_alignment_annotations__331_PSG.tar.gz`
  `jalview_alignment_annotations__416_aPSG.tar.gz`
- Jalview sequence annotations
  `jalview_sequence_annotations__331_PSG.tar.gz`
  `jalview_sequence_annotations__416_aPSG.tar.gz`

Jalview annotation files contain annotations for the positively selected residues (PSR), as well as exon coordinates mapped to protein sequences. They can be loaded together with the correct corresponding alignment using (on Mac):

```
Java     -Djava.ext.dirs=/Applications/Jalview/lib/
         -cp /Applications/Jalview/jalview.jar jalview.b.Jalview
         -open           <ENSEMBL_ID>__cds.prank-codon-guidance-tcs-masked-species-
sorted.aln.translated.fa
         -annotations <ENSEMBL_ID>.jalview_aln_feature
         -features <ENSEMBL_ID>.jalview_seq_feature
```

Configuration files for PAML codeml

- Inference of reference phylogenetic trees
  `codeml_M0_F3X4_tree.ctl`
  `codeml_M0_F61_tree.ctl`
- Inference of positive selection
  `codeml_M1avM2a_F3X4__large-scale-analysis__template.ctl`
  `codeml_M1avM2a_F61__large-scale-analysis__template.ctl`
  `codeml_M7vM8_F3X4__large-scale-analysis__template.ctl`
  `codeml_M7vM8_F61__large-scale-analysis__template.ctl`

Phylogenetic trees

- codeml reference phylogenetic trees
  `codeml_M0_tree__unrooted_tree__F3X4.tre`
  `codeml_M0_tree__unrooted_tree__F61.tre`
- Other phylogenetic trees
  `Ensembl78__9primates__with_taxon_id__unrooted.tre`
  `Perelman_et_al__9primates__unrooted.tre`
  `RAxML_bestTree__9primates__unrooted.tre`

GUIDANCE source code fix for running PRANK

- `guidance_prank_fix.pdf`
- `Guidance__prank_fix.pm`

Supplementary Text

Text S1. Introduction to molecular adaptation and inference of positive selection from comparative evolutionary analyses.

Molecular adaptation caused by positive selection

Acquired phenotypic traits can have three broad types of effects: (i) the trait has no effect on the ability to survive and reproduce (fitness) of the individual carrying it (i.e. the trait will be selectively neutral), (ii) the trait has a negative effect on fitness and its prevalence in the population should decrease (a process referred to as negative or purifying selection), or (iii) the trait has a positive effect on fitness and its prevalence in the population should increase (positive Darwinian selection). The actual ability of natural selection (24) to change the prevalence of a phenotypic trait in a population depends on a variety of factors, including the magnitude of the fitness advantage or disadvantage caused by the trait and the effective size of the population.

The neutral theory of molecular evolution (25) proposes that the majority of evolutionary changes at the molecular level are deleterious and therefore purged by negative selection, while most of the remaining mutations are neutral rather than beneficial. According to this view, beneficial mutations leading to positive selection are rare. Therefore, the majority of observed molecular variation in populations has been proposed to be the result of random sampling of selectively neutral or nearly neutral genetic variants (genetic drift). However, despite their proposed rarity, mutations creating advantageous phenotypes can be a great source of insights into the adaptive evolutionary processes governing the development of species and populations.

Changes in the DNA of a species (or other heritable features, e.g. epigenetic changes) can result in variety of molecular traits that might influence cellular and organismal phenotypes, and hence fitness (though the contribution of individual variations to fitness tends to be small (26)). For instance, mutations in the regulatory elements of the genome influence transcription and gene expression, while mutations to the coding sequence of genes (cDNA) can lead to changes in the protein structure. Since a large part of cellular functionality is achieved through proteins, such protein-changing mutations can have direct consequences for cellular phenotype. Therefore, many studies on molecular adaptation focus on the protein-coding

regions of the genome. Statistical methods have been developed (i) for detecting which genes are likely to have evolved under positive selection, (ii) for inferring which codons within those genes are responsible for their positive selection signatures, and (iii) for finding out whether specific lineages or sequences underwent episodes of adaptive change (27).

Inferring genes and codons under positive selection by d_N/d_S-based maximum likelihood methods

Because of the degeneracy in the genetic code (i.e. all amino acids except methionine and tryptophan can be encoded by multiple codons), some mutations in the cDNA cause changes to the protein sequence (nonsynonymous substitutions), while other mutations do not change the protein sequence (silent, synonymous substitutions). An excess of nonsynonymous substitutions (normalized by the total possible nonsynonymous sites, d_N) over synonymous substitutions (normalized by the total possible synonymous sites, d_S) has proved to be a good indicator of positive selection acting on a gene (28). Interpretation of this $\omega = d_N/d_S$ ratio (also known as K_a/K_s) is based on the assumption that silent substitutions are neutral and fixed by random genetic drift. Therefore, d_S can serve as a baseline for the expected rate of (neutral) nonsynonymous substitution. Thus, the mode of selection acting on a gene or a specific codon can be inferred from the value for ω as follows:

- $\omega = 1$; equal rates of d_N and d_S; nonsynonymous substitutions are neutral on average
- $\omega < 1$; lower rates of d_N than d_S; part of the nonsynonymous substitutions are deleterious and purged by negative/purifying selection
- $\omega > 1$; higher rates of d_N than d_S; part of the nonsynonymous substitutions are beneficial and retained by positive selection

It should be noted that estimates of ω for an entire gene represents an average of the d_N/d_S ratios of all codons, which are likely to be heterogeneous and many of which are probably strongly conserved. Thus, d_N/d_S-based methods tend to require a strong signal across multiple lineages in order to identify positive selection.

Several evolutionary phenomena and assumptions confound d_N/d_S estimates. First, synonymous substitutions are not always neutral as they can influence for example mRNA stability and splicing, and can even influence the final protein, perhaps by influencing co-translational folding (29). Furthermore, (i) nucleotide substitutions show a higher rate of transitions than transversions, (ii) different codons encoding the same amino acid are not used

in equal amounts (biased codon usage), (iii) multiple substitutions may have occurred over time at the same position, other than the differences present between the final observed sequences, and (iv) different sequences (species) have undergone different divergence times between them, which influences expected amounts of variation between sequences (28, 30). Analyses of d_N/d_S work best for sequences that are sampled across an appropriate, 'medium' evolutionary distance: they should be distant enough to show some variation, but not too divergent to limit the effects of multiple substitutions occurring at the same site and prevent the associated loss of information (31).

Maximum likelihood (ML) methods have been shown to best account for these confounding factors and achieve reliable d_N/d_S estimates based on evolutionary models. They work by simultaneously estimating the combination of parameters (the d_N/d_S ratio ω, the transition/transversion rate ratio κ, codon frequencies π) that best explains the sequence data (in the form of a multiple sequence alignment), given the divergence time t between sequences (i.e. expected amount of change, as represented by the branch lengths of a phylogenetic tree (28)). The codeml program in the PAML software package ('Phylogenetic Analysis by Maximum Likelihood') is most popular (32) for detecting positive selection in multiple sequence alignments of protein coding sequences.

The method explores many combinations of values for parameters ω, κ, and π and for each combination constructs a matrix of codon transition probabilities over time t, $P(t)$ (28). The value for time t between two sequences (divergence) can either also be explored as a free parameter, or can be fixed based on external data (branch lengths in a provided phylogenetic tree). The probability of one codon replacing another represents a Markov process of codon substitution (33), which only depends on the current identity of the codons and is independent of their history. Each codon transition probability state is then used to calculate the likelihood that the corresponding combination of parameters would have given rise to the data in the sequence alignment (i.e. the codons observed across the sequences). For each codon position (referred to as a 'site') in the alignment, the likelihood that a particular transition probability matrix gave rise to the observed codon configuration across the sequences is obtained by multiplying the probabilities of substituting one codon with another, at each part of the tree, summing over all possible ancestral states (34). Under the assumption that sites evolve independently from each other, the likelihood for the full alignment is obtained by

multiplying the likelihoods per site. Maximizing the full-alignment likelihood eventually leads to the combination of parameters, including ω, κ, and π, that best fit the data.

Genes can be assessed for different 'models' of natural selection by varying the range of values that ω is allowed to take in the maximum likelihood calculations. To test for positive selection, one can ask whether a null model (or neutral model; ω is only allowed to range from 0 to 1) better fits the data than an alternative model (or selection model; ω is allowed to take values larger than 1) (28, 35). The likelihood ratio test assesses whether the alternative model better explains the data than does the null model, by comparing twice the log-likelihood difference between the two models to a chi-square test distribution (LRT = 2×(lnL[selection model] – lnL[neutral model]). A statistically significant better fit of the alternative model suggests that positive selection may have acted on the gene.

Various neutral and selection site models have been defined (28, 35). They differ in the distributions from which ω is sampled during the maximum likelihood parameter estimation. Two model comparisons have the best power and are commonly performed:

(i) M1a (neutral) vs. M2a (selection)

Model M1a fits two ω site classes in proportions p_0 and $p_1 = 1 - p_0$ with $0 < \omega_0 < 1$ (purifying selection) and $\omega_1 = 1$ (neutral). Model M2a adds a proportion $p_2 = 1 - p_0 - p_1$ of sites with $\omega_2 > 1$ (positive selection).

(ii) M7 (beta) vs. M8 (beta&ω)

Model M7 fits ω between 0 and 1 as a beta distribution, the shape of which (e.g. L-, U-, ∩-, J-shaped) depends on beta distribution parameters *p* and *q*, which are estimated in the ML calculation. In model M8, a proportion p_0 of sites have ω drawn from a beta distribution and a proportion $p_1 = 1 - p_0$ of sites have a single $\omega_1 > 1$.

Genes that shown evidence of having evolved under positive selection according to maximum likelihood analysis can then be studied to determine which sites (codons, and thus residues/amino acids) are subject to selection. The ML procedure results in an estimated distribution of ω values across the gene as well as the estimated proportions with which these ω values occur. Inference of which sites evolved under positive selection follows a Bayesian approach (Bayes empirical Bayes), which uses the ML estimates for the values and proportions of ω classes as prior probabilities for each site in the alignment (28, 36). The prior probabilities are updated according to the data at a site in the alignment (the observed codons

across the sequences), to arrive at site-specific posterior probabilities for each class of ω values: $P_{posterior}(\omega \mid data) = P_{prior}(\omega) \cdot P(data \mid \omega) / P(data)$. The ω class that maximizes the posterior probability is the most likely class for the site. A high posterior probability (typically >0.90 or >0.95) for the $\omega > 1$ class suggests that the site in question evolved under positive selection.

Detecting positive selection from within-species variation

Besides between-species sequence variation approaches such as d_N/d_S, within-species variation can also be exploited to study molecular adaptation. Many such intraspecies approaches are based on population genetics statistics around the concept of selective sweeps. This model presumes that recent strongly advantageous mutations become rapidly fixed in a population or species (37-39). Due to genetic hitchhiking, fixation of the mutation would cause genetically linked alleles (which usually locate to the region surrounding the mutation) to also increase in frequency or become fixed, while alleles that are not linked to the mutation will decrease in frequency or be lost. Thus, selective sweeps, compared to neutrality, generally result in a decrease in genetic diversity across a larger genomic region surrounding a favorable mutation. The lack of sequence diversity appears for instance as an excess of rare alleles and increased linkage disequilibrium in the region (40).

A final approach for detecting adaptation at the molecular level makes use of both inter- and intra-species variation. The McDonald-Kreitman test compares levels of variation occurring within species (referred to as polymorphism) to levels of variation between species (referred to as divergence)(41). This allows an estimation of how much of the variation between species is driven to fixation within species. If all substitutions are neutral, both types of variation should be similar, while an excess of nonsynonymous variation between species compared to within species indicates positively selected variants driven to fixation (41, 42). In contrast to this model, we find that many of the positively selected positions in primates (with high between-species d_N/d_S) show an elevated rather than a reduced level of nonsynonymous over synonymous mutations within humans, arguing against fixation of these variations and in favor of ongoing change (see **Main Text**).

Supplementary References

1. Perelman,P., Johnson,W.E., Roos,C., Seuánez,H.N., Horvath,J.E., Moreira,M.A.M., Kessing,B., Pontius,J., Roelke,M., Rumpler,Y., *et al.* (2011) A molecular phylogeny of living primates. *PLoS Genet.*, **7**, e1001342.
2. Yates,A., Akanni,W., Amode,M.R., Barrell,D., Billis,K., Carvalho-Silva,D., Cummins,C., Clapham,P., Fitzgerald,S., Gil,L., *et al.* (2016) Ensembl 2016. *Nucleic Acids Res.*, **44**, D710–6.
3. Oliveros,J.C. Oliveros, J.C. (2007-2015) Venny. An interactive tool for comparing lists with Venn's diagrams. http://bioinfogp.cnb.csic.es/tools/venny/index.html.
4. Rosenbloom,K.R., Armstrong,J., Barber,G.P., Casper,J., Clawson,H., Diekhans,M., Dreszer,T.R., Fujita,P.A., Guruvadoo,L., Haeussler,M., *et al.* (2015) The UCSC Genome Browser database: 2015 update. *Nucleic Acids Res.*, **43**, D670–81.
5. Lander,E.S., Linton,L.M., Birren,B., Nusbaum,C., Zody,M.C., Baldwin,J., Devon,K., Dewar,K., Doyle,M., FitzHugh,W., *et al.* (2001) Initial sequencing and analysis of the human genome. *Nature*, **409**, 860–921.
6. Venter,J.C., Adams,M.D., Myers,E.W., Li,P.W., Mural,R.J., Sutton,G.G., Smith,H.O., Yandell,M., Evans,C.A., Holt,R.A., *et al.* (2001) The sequence of the human genome. *Science*, **291**, 1304–1351.
7. Chimpanzee Sequencing and Analysis Consortium (2005) Initial sequence of the chimpanzee genome and comparison with the human genome. *Nature*, **437**, 69–87.
8. Scally,A., Dutheil,J.Y., Hillier,L.W., Jordan,G.E., Goodhead,I., Herrero,J., Hobolth,A., Lappalainen,T., Mailund,T., Marquès-Bonet,T., *et al.* (2012) Insights into hominid evolution from the gorilla genome sequence. *Nature*, **483**, 169–175.
9. Locke,D.P., Hillier,L.W., Warren,W.C., Worley,K.C., Nazareth,L.V., Muzny,D.M., Yang,S.-P., Wang,Z., Chinwalla,A.T., Minx,P., *et al.* (2011) Comparative and demographic analysis of orang-utan genomes. *Nature*, **469**, 529–533.
10. Carbone,L., Harris,R.A., Gnerre,S., Veeramah,K.R., Lorente-Galdos,B., Huddleston,J., Meyer,T.J., Herrero,J., Roos,C., Aken,B., *et al.* (2014) Gibbon genome and the fast karyotype evolution of small apes. *Nature*, **513**, 195–201.
11. Rhesus Macaque Genome Sequencing and Analysis Consortium, Gibbs,R.A., Rogers,J., Katze,M.G., Bumgarner,R., Weinstock,G.M., Mardis,E.R., Remington,K.A., Strausberg,R.L., Venter,J.C., *et al.* (2007) Evolutionary and biomedical insights from the rhesus macaque genome. *Science*, **316**, 222–234.
12. Marmoset Genome Sequencing and Analysis Consortium (2014) The common marmoset genome provides insight into primate biology and evolution. *Nat. Genet.*, **46**, 850–857.
13. Calvo,S.E., Clauser,K.R. and Mootha,V.K. (2016) MitoCarta2.0: an updated inventory of mammalian mitochondrial proteins. *Nucleic Acids Res.*, **44**, D1251–7.
14. Xu,F., Ackerley,C., Maj,M.C., Addis,J.B.L., Levandovskiy,V., Lee,J., Mackay,N., Cameron,J.M. and Robinson,B.H. (2008) Disruption of a mitochondrial RNA-binding protein gene results in decreased cytochrome b expression and a marked reduction in ubiquinol-cytochrome c reductase activity in mouse heart mitochondria. *Biochem. J.*, **416**, 15–26.
15. Khalimonchuk,O., Bestwick,M., Meunier,B., Watts,T.C. and Winge,D.R. (2010) Formation of the redox cofactor centers during Cox1 maturation in yeast cytochrome oxidase. *Mol. Cell. Biol.*, **30**, 1004–1017.
16. Guerrero-Castillo,S., Baertling,F., Kownatzki,D., Wessels,H.J., Arnold,S., Brandt,U. and Nijtmans,L. (2017) The Assembly Pathway of Mitochondrial Respiratory Chain Complex I. *Cell Metab.*, **25**, 128–139.
17. Fiedorczuk,K., Letts,J.A., Degliesposti,G., Kaszuba,K., Skehel,M. and Sazanov,L.A. (2016) Atomic structure of the entire mammalian mitochondrial complex I. *Nature*, **538**, 406–410.
18. Spåhr,H., Rozanska,A., Li,X., Atanassov,I., Lightowlers,R.N., Chrzanowska-Lightowlers,Z.M.A., Rackham,O. and Larsson,N.-G. (2016) SLIRP stabilizes LRPPRC via an RRM-PPR protein interface. *Nucleic Acids Res.*, **44**, 6868–6882.
19. Karpenahalli,M.R., Lupas,A.N. and Söding,J. (2007) TPRpred: a tool for prediction of TPR-, PPR- and SEL1-like repeats from protein sequences. *BMC Bioinformatics*, **8**, 2.
20. Gagnon,M.G., Lin,J. and Steitz,T.A. (2016) Elongation factor 4 remodels the A-site tRNA on the

ribosome. *Proc. Natl. Acad. Sci. U.S.A.*, **113**, 4994–4999.

21. Sologub,M., Litonin,D., Anikin,M., Mustaev,A. and Temiakov,D. (2009) TFB2 is a transient component of the catalytic site of the human mitochondrial RNA polymerase. *Cell*, **139**, 934–944.
22. Alonso,R., Salavert,F., Garcia-Garcia,F., Carbonell-Caballero,J., Bleda,M., Garcia-Alonso,L., Sanchis-Juan,A., Perez-Gil,D., Marin-Garcia,P., Sanchez,R., *et al.* (2015) Babelomics 5.0: functional interpretation for new generations of genomic data. *Nucleic Acids Res.*, **43**, W117–21.
23. Huang,D.W., Sherman,B.T. and Lempicki,R.A. (2009) Systematic and integrative analysis of large gene lists using DAVID bioinformatics resources. *Nat Protoc*, **4**, 44–57.
24. Darwin,C. (1859) On the Origin of Species by Means of Natural Selection, Or, The Preservation of Favoured Races in the Struggle for Life J. Murray, London.
25. Kimura,M. (1983) The neutral theory of molecular evolution Cambridge University Press, Cambridge.
26. Eyre-Walker,A. and Keightley,P.D. (2007) The distribution of fitness effects of new mutations. *Nat. Rev. Genet.*, **8**, 610–618.
27. Yang,Z. (2008) Adaptive Molecular Evolution. In *Handbook of Statistical Genetics*. John Wiley & Sons, Ltd, pp. 375–406.
28. Yang,Z. and Bielawski,J. (2000) Statistical methods for detecting molecular adaptation. *Trends Ecol Evol*, **15**, 496–503.
29. Chamary,J.V., Parmley,J.L. and Hurst,L.D. (2006) Hearing silence: non-neutral evolution at synonymous sites in mammals. *Nat. Rev. Genet.*, **7**, 98–108.
30. Hurst,L.D. (2002) The Ka/Ks ratio: diagnosing the form of sequence evolution. *Trends Genet.*, **18**, 486.
31. Anisimova,M., Bielawski,J.P. and Yang,Z. (2001) Accuracy and power of the likelihood ratio test in detecting adaptive molecular evolution. *Mol. Biol. Evol.*, **18**, 1585–1592.
32. Yang,Z. (2007) PAML 4: phylogenetic analysis by maximum likelihood. *Mol. Biol. Evol.*, **24**, 1586–1591.
33. Goldman,N. and Yang,Z. (1994) A codon-based model of nucleotide substitution for protein-coding DNA sequences. *Mol. Biol. Evol.*, **11**, 725–736.
34. Felsenstein,J. (1981) Evolutionary trees from DNA sequences: a maximum likelihood approach. *J. Mol. Evol.*, **17**, 368–376.
35. Yang,Z., Nielsen,R., Goldman,N. and Pedersen,A.M. (2000) Codon-substitution models for heterogeneous selection pressure at amino acid sites. *Genetics*, **155**, 431–449.
36. Yang,Z., Wong,W.S.W. and Nielsen,R. (2005) Bayes empirical bayes inference of amino acid sites under positive selection. *Mol. Biol. Evol.*, **22**, 1107–1118.
37. Fu,W. and Akey,J.M. (2013) Selection and adaptation in the human genome. *Annu Rev Genomics Hum Genet*, **14**, 467–489.
38. Messer,P.W. and Petrov,D.A. (2013) Population genomics of rapid adaptation by soft selective sweeps. *Trends Ecol Evol*, **28**, 659–669.
39. Sabeti,P.C., Schaffner,S.F., Fry,B., Lohmueller,J., Varilly,P., Shamovsky,O., Palma,A., Mikkelsen,T.S., Altshuler,D. and Lander,E.S. (2006) Positive natural selection in the human lineage. *Science*, **312**, 1614–1620.
40. Suzuki,Y. (2010) Statistical methods for detecting natural selection from genomic data. *Genes Genet. Syst.*, **85**, 359–376.
41. Fay,J.C. (2011) Weighing the evidence for adaptation at the molecular level. *Trends Genet.*, **27**, 343–349.
42. Eyre-Walker,A. (2006) The genomic rate of adaptive evolution. *Trends Ecol Evol*, **21**, 569–575.
